# Ten year maturation period in a level-I trauma center, a cohort comparison study

**DOI:** 10.1007/s00068-016-0722-1

**Published:** 2016-09-15

**Authors:** A. M. K. Harmsen, G. F. Giannakopoulos, M. Terra, E. S. M. de Lange de Klerk, F. W. Bloemers

**Affiliations:** 1 Department of Surgery, VU University Medical Center, De Boelelaan 1117, P.O. Box 7057, 1081 HV Amsterdam, The Netherlands; 2 Department of Surgery, MC Slotervaart, Louwesweg 6, 1066 EC Amsterdam, The Netherlands; 3 Department of Epidemiology and Biostatistics, VU University Medical Center, De Boelelaan 1117, 1081 HV Amsterdam, The Netherlands

**Keywords:** Trauma, Center, Maturation, Outcome, Emergency medical services

## Abstract

**Purpose:**

Many changes have been made to improve trauma care. Improved trauma team response and usage of a hybrid resuscitation room are examples of how this trauma center has developed. The aim was to assess how the outcome of the trauma population was influenced by the maturation.

**Methods:**

A cohort comparison, between June 2004–July 2005 and 2014, was performed. All adult trauma patients with an Injury Severity Score (ISS) >15 were included. Variables collected were: patient demographics, mechanism of trauma, total prehospital time, pre- and inhospital trauma scores, vital signs, blood values and interventions, and physician staffed helicopter emergency medical services (P-HEMS) involvement and outcome.

**Results:**

From June 2004 to July 2005 219, patients were admitted, and for the year 2014, this was 282 patients. The 2014 cohort was significantly older (mean age of 53.6 ± 23.8 vs 45.6 ± 22.7 years). The mean RTS did not differ. P-HEMS assists increased to 116 (13.5 %). The number of CT scans, blood transfusion, and acute trauma surgical interventions decreased. Mean LOS, ICU admission, and ICU LOS did not differ. The mortality rate, however, decreased by 7.0 %, observed and predicted survival was significantly different in favour of the 2014 cohort, with a Z-score of 4.25.

**Conclusion:**

An increase in age is seen, though trauma scores remain comparable. The number of blood products transfused and acute trauma surgical interventions performed declines. Mortality significantly decreased and a significant difference in observed and predicted survival is seen. Showing improved trauma care in our hospital, in favour of the second period.

## Introduction

The number of deaths due to injury has increased over the last decades. Nowadays, over 15,000 people die each day as a result of injury. It accounts for nine per cent of the World’s deaths annually [1]. Most of these deaths are traffic or violence related, which are predictable and largely preventable causes of injury. Consequently, many efforts have been made to reduce these numbers by improving the quality of trauma care and subsequently improving trauma patient outcome. Prehospital trauma care has evolved significantly, amongst others due to: the implementation of trauma centers, more protocoled work in the dispatch centers and on the ambulances, implementation of mass transfusion protocol, and by extending prehospital trauma care with the physician staffed helicopter emergency medical services (P-HEMS). The P-HEMS supplements the prehospital trauma life support performed by the EMS with advanced trauma care, Dutch P-HEMS can perform procedures, such as rapid sequence intubation, administer advanced pain medication, inotropes, vasopressors, and other medication. Moreover, an P-HEMS team can perform invasive surgical interventions [2]. These prehospital intervention strategies are amongst others of influence on the patient’s hemodynamic parameters and survival. One can imagine that this affects the characteristics of the trauma patient population that does not die in the prehospital setting and is brought to a trauma center. Furthermore, the inhospital triage systems have changed as well. This level-1 trauma center now uses a two-tiered trauma team activation model. Trauma team activation is based on specific criteria concerning patients’ vitals, mechanism of injury, and type of injuries sustained. A divide is made in the dispatch of a complete trauma team or a selective trauma team. Other inhospital changes are the availability of a hybrid emergency resuscitation room [3] and implementation of a massive transfusion protocol [4, 5]. The objective of all these efforts is to reduce morbidity and mortality for those who do suffer an injury and to allocate resources properly. This to reduce over and under triage. Earlier studies evaluating the maturation of level-1 trauma centers show an improvement in outcome [6–8]. It is of great importance to evaluate the transformations made to improve trauma care. Therefore, the aim of this observational cohort comparison study was to assess, how the outcome of the trauma population treated in this level-1 trauma center was influenced by the maturation of the pre- and inhospital trauma care, as we hypothesize an improvement in survival could be seen. Furthermore, we were interested to see changes in trauma mechanisms, occurrence of injuries and treatment at our trauma center.

## Methods

### Data collection

We performed an observational cohort comparison study. Data were collected for all adult trauma patients with an injury severity score (ISS) above 15, who were admitted to a level-1 trauma center, in 2014. This cohort was compared to a historical cohort likewise comprising of trauma patients admitted with an ISS >15 between June 2004 and July 2005. For the purpose of this study, data were collected from two databases. (1) Data for both cohorts were obtained from the National Regional Trauma Database (NRTD) located at the trauma center. The NRTD is the national trauma registry, implemented by the Dutch ministry of health, and governed by the National network acute care (NNAC). The data collection and coding is locally done by dedicated database managers, and data verification and validation is done by the NNAC. It is a registration system for all trauma patients in the Netherlands and we retrieved only the data for the patients admitted to this level-1 trauma center. (2) Furthermore, data were retrieved from the inhospital patient registration system. The NRTD was searched using a query in the ISS field box-selecting patients with an ISS above 15 to include only the severely injured and to calculate ISS that the updated version 1998 of AIS was used [9]. Patients were matched to the Inhospital patient registration system using a personal code. Variables that were retrieved were: patient demographics, details on the trauma mechanism, duration of prehospital time intervals, prehospital trauma scores, prehospital vital signs, P-HEMS, emergency room (ER) vital signs, ER trauma scores, ER arterial blood gas values, intoxication status, emergency intervention, transfusion of blood products, length of stay (LOS), number of days of intensive care unit (ICU) admission, and inhospital mortality. Patients were excluded if dead on arrival, incomplete prehospital or inhospital data or patients who were relocated to a different hospital.

### Analysis

The statistical data analysis was performed using the SPSS 21.0 Statistical Analysis program (SPSS Inc., Chicago, IL).

Continuous data are reported as mean with the standard deviations (SD) for normally distributed data and as median [5–95 percentiles with inter quartile rage (IQR)] for not normally distributed data. The ISS is calculated from the abbreviated injury scale for each body region and represents the severity of the injuries sustained. The probability of survival (Ps) was determined using the trauma score-injury severity score (TRISS) formulae [10] using variables from both databases, the same for both cohorts. The TRISS determines the Ps based on a patient’s ISS, RTS, age, and type of trauma (blunt or penetrating trauma). Differences between the two groups with respect to mechanism of injury were assessed using the Chi-square tests with Bonferroni correction, and the significance of statistical differences was attributed to a two tailed *p* value of <0.05. Student’s *t* test and Chi-square tests were used to test for differences in the duration of hospital stay, ICU stay, and mortality for the two cohorts, and statistical significance was set at *α* = 0.05. Logistic regression analysis was used to compare survival in both cohorts, adjusted for age and ISS score. The* Z* statistic was calculated to evaluate the difference between predicted and observed survival. A negative* Z* statistic indicates the observed survival to be lower than the predicted survival, a positive* Z* statistic indicates the observed survival to be higher than the predicted survival, and *p* values <0.05 were considered statistically significant [11, 12].

## Results

From June 2004 to July 2005 219, patients were admitted with an ISS >15. For the year 2014, this was 282 patients with an ISS >15. Patient characteristics for both cohorts can be found in Table 1. The 2014 cohort was significantly older than the 2004–2005 cohort with a mean age of 53.6 ± 23.8 years (median of 55, 0 years) versus a mean age of 45.6 ± 22.7 years (median of 43, 6 years)(*p* < 0.001). The male/female ratio and the mean ISS score did not change from one study period to the next. The mean RTS and GCS likewise did not differ for both cohorts. Compared to the first period, the number of P-HEMS assists increased by 13.5 % (*p* < 0.001). The total prehospital time did not differ. RTS and GCS on arrival at the emergency room remained unchanged as did pH and Hb levels. Additional biochemical parameters can be seen in Table 1. When reviewing the mechanism of injury, the proportion of motor vehicle crashes significantly declined from 38 (17 %) in 2004–2005 to 20 (7 %) in 2014 using the Chi-square tests with Bonferroni correction. The category “other” mechanisms increased significantly from zero cases in 2004–2005 to 21 cases in 2014. The proportion of further mechanisms of trauma did not differ significantly from one study period to the next. When grouped together the number of penetrating injuries differed significantly, there were three (1.5 %) penetrating injuries in 2004–2005 and there were 14 (5.0 %) penetrating injuries over 2014. An overview of the mechanisms of injury is presented in Table 2. An overview of all inhospital interventions is presented in Table 3. A significant decrease is seen in the number of intubations in the 2014 cohort versus for 2004–2005 (*p* = 0.008), the same as for the number of CT scans (*p* = 0.044). The number of packed cells (PC) and fresh frozen plasma (FFP) transfused within 24 h of arrival likewise decreased significantly, for PC this was 1.0 for 2014 versus 2.7 for 2004–2005 (*p* = 0.011) and for FFP this was 0.4 for 2014 versus 1.0 for 2004–2005 (*p* = 0.001). When looking at the total amount of blood products transfused within the entire period of admission, the number of packed cells (PC) and fresh frozen plasma (FFP) decreased significantly, the number of total acute interventions lessened by 14.3 % (*p* = 0.001), and the number of acute trauma surgical interventions lessened by 8.8 % (*p* = 0.005). However, the number of acute angio-interventional radiology procedures increased significantly from two in the 2004–2005 cohort to ten in the 2014 cohort (*p* = 0.002). Focusing on the different outcome measures, mean LOS was 14 ± 16 days for the 2014 cohort which is not significantly different to the 20 ± 29 days for the 2004–2005 cohort. ICU admission and length of ICU admission did not differ from one study period to the other. The cumulative mortality proportion, however, decreased by 7.0 % (*p* = 0.043). The mean probability of survival did not differ for both periods. After adjusting for ISS and age using logistic regression analysis, the 2014 cohort had an OR of 1.9 for survival (95 % CI 1.14–3.3, *p* = 0.014) compared to the 2004–2005 cohort. The mean probability of survival was not significantly different. The difference in observed and predicted survival, however, was significantly different in favour of the second period. The Z-score of the 2014 period, *Z* = 4.25 (*p* = 0.002) showed a significant positive difference between the observed and predicted survivals. Further outcome measures are depicted in Table 4.

**Table 1:** Patient characteristics

	2004–2005	2014	*p* value
Patients, *n*	219	282	n.a.
Male, *n* (%)	145 (66.2)	195 (69.1)	0.485
Mean age	45.6 ± 22.7	53.6 ± 23.8	<0.001
ISS	24.0 ± 8.1	23.2 ± 7.8	0.267
Prehospital RTS	6.4 ± 2.0	6.8 ± 2.3	0.190
ER RTS	6.5 ± 1.8	6.5 ± 2.0	0.844
Prehospital GCS median (IQR)	14 (6–15)	14 (7–15)	0.935
ER GCS median (IQR)	14(4–15)	14 (3–15)	0.961
P-HEMS presence, *n* (%)	61 (27.9)	116 (41.4)	0.001
OST			
TPT	44.7 (15.2)	45.4 (12.6)	0.063
Intubation	n.r	38 (13.5)	
ER pH	7.33 (0.1)	7.4 (0.1)	0.055
ER BE	−2.7 (2.9)	−0.7 (3.7)	<0.001
ER pO_2_	202.0 (110.5)	126.7 (99.8)	<0.001
ER Hb	7.7 (1.5)	7.5 (1.6)	0.272

*N* number, *n.a.* not applicable, *ISS* injury severity score, *RTS* revised trauma score, *ER* emergency room, *IQR* inter quartile range, *CGS* Glasgow coma score, *P-HEMS* physician staffed helicopter emergency medical service, *OST* on scene time, *TPT* total prehospital time, *n.r* not recorded, *pH* numeric scale for acidity, *BE* base excess in mmol/L, *pO*_*2*_ partial pressure of Oxygen in mm Hg, *Hb* hemoglobin levels in mmol/L

**Table 2:** Mechanisms of injury

	2004–2005	2014
Fall of height >2 meters	71	69
Same level fall	13	39
Pedestrian injured	21	13
Cycling crash	32	45
Scooter/motor vehicle crash	26	39
Car vehicle crash	38*	20*
Crash involving large vehicle	1	0
Drowning/suffocation	0	6
Burns	0	3
Violence	7	6
Crush injury	7	8
Penetrating injury *n* (%)	3	14º
Gunshot injury	1	5
Stab injury	2	9
Other	0*	21*

º Significant difference using the Chi-square tests

* Significant difference using the Chi-square tests with Bonferroni correction

**Table 3:** Interventions

	2004–2005	2014	*p* value
Intubation, *n* (%)	38 (17.4)	26 (9.3)	0.008
CT-scan, *n* (%)	201 (93.1)	247 (87.6)	0.044
PC 24 h	4 (2–9)	2 (2–5)	0.011
FFP 24 h	4 (3–7)	3.5 (2–6)	0.001
Pl 24 h	1 (1–2)	1 (1–2)	0.128
PC total	6 (2–10)	3 (2–6)	<0.001
FFP total	4 (3–7)	3 (2–6)	<0.001
Pl total	1 (1–2)	2 (1–2)	0.109
Acute intervention	85 ± 38.8	75 ± 26.6	0.001
Trauma, *n* (%)	41 (18.7)	28 (9.9)	0.005
Neuro surgery, *n* (%)	44 (20.1)	61 (21.6)	0.674
Maxillofacial, *n* (%)	4 (1.8)	1 (0.4)	0.100
Angio intervention, *n* (%)	2 (1.0)	10 (3.5)	0.002

*N* number, *CT-scan* computed tomography scan, *PC* packed cells, *FFP* fresh frozen plasma, *Pl* platelets

**Table 4:** Outcome measures

	2004–2005	2014	*p* value
Median LOS (IQR)	11 (4–25)	9 (4–18)	0.162
ICU admission, *n* (%)	135 (61.6)	183 (64.9)	0.454
Median ICU LOS (IQR)	2 (0–5)	2 (0–5)	0.376
Inhospital mortality, *n* (%)	47 (21.5)	41 (14.5)	0.043
TRISS			
Mean Ps	0.79	0.78	0.952
Expected Mortality	16.3 %	16.0 %	
Observed mortality	0.27	0.19	
*Z* score	−0.48 (*p* = 0.31)	4.25 (*p* < 0.0002)	

*LOS* length of stay, *IQR* inter quartile range, *N* number, *ICU* intensive care, *SD* standard deviation, *ICU* intensive care unit, *TRISS* trauma and injury severity score

## Discussion

This study compares two cohorts of severely injured patients admitted to an urban level-1 trauma center almost 10 years apart. The data presented show that impressive changes have occurred over this period. The goal of a trauma center is to enhance and optimize care of the severely injured patient [13]. To do so, several changes in trauma care have been made, amongst others renewing of the emergency resuscitation room [3], the implementation of an improved two-tiered triage system, and the implementation of a massive transfusion protocol [4, 5]. The aim of this observational cohort study was to evaluate the outcome of the major trauma patient in a level-1 trauma center after all these alterations. We compared two cohorts, the first covering the period from June 2004 to July 2005 and the second covering the entire year of 2014. This was done because from January 2015 on the ISS scores are calculated differently and, therefore, no longer comparable to the historical cohort [14]. Besides the increase in the total number of admitted patients with an ISS >15, the 8 year increase in the mean age of the second cohort is remarkable. This could be due aging of the entire population. Since trauma is a disease process that affects all age groups, elderly make up one of the fastest growing segments. Furthermore, mortality and morbidity are influenced by age, physical condition, and comorbidities and, therefore, elderly might be triaged to a higher level trauma center more quickly than their younger equivalents [15, 16]. The 13 % increase in P-HEMS presence in the prehospital phase is most likely due to the fact that in 2009, the Dutch government granted 24/7 coverage for the four Dutch P-HEMS crews, allowing them to also do night flights [17, 18]. The increased prehospital presence of the P-HEMS is very likely to lead to an increased prehospital intubation rate [19]. This could in turn explain the decreased ED intubation rate. In the 2014 cohort, significantly less acute interventions are performed, especially less trauma surgical intervention. This could be due to increased sensitivity of the CT-imaging, allowing the surgeon to treat clinically significant injuries more properly and thus treating patients more frequent with a non-surgical approach [20, 21]. The decline in acute trauma surgical interventions can also be attributed to the increased usage of angio interventions. Angio-embolisation has gained in popularity in identification and arresting bleeding in trauma patients, decreasing the need for surgical intervention [22, 23]. In addition, for the 2014 cohort, a decrease is seen in the usage of blood products. Especially, packed cells and fresh frozen plasma, both in the acute setting as for the entire duration of admission, are transfused less than in the 2004–2005 period. One could think that the usage of the MTP is reason for this decline [24]. The decline in crude mortality rate can be attributed to many factors, as outcome is greatly affected by time to definite care, quality of care, injury severity, and patient factors [25, 26]. Total prehospital time and trauma scores did not differ for both periods; however, the 2014 cohort was significantly older. This is correlated with the extensive comorbidity [27]. Though even in the absence of comorbidity, age is a known risk factor of adverse outcomes independent of other patient characteristics [28, 29]. We can conclude that progress has been made in trauma care of this level-1 trauma center, as we observed a significant difference in mortality rates and in observed and predicted survival in favour of the second period, with a Z-score of 4.25, indicating significantly more survivors in our institution than expected. The improvement in survival can be attributed to the improvement in the quality of care over time as well as the changes that this trauma center has undergone. Likewise, it is proven that a surgeons experience with the trauma center or system positively influences outcome, this attributes to the improvement over time [30].

A limitation of this study it its retrospective nature. As is the usage of trauma registries, this was the main reason for missing ISS or RTS scores. Another limitation is that we were only able to analyse variables that were collected during both periods, minimizing the comparisons to be made, because not all parameters were scored during the first cohort. Furthermore, we only assessed inhospital outcome, one could deliberate on outcome 1 year after trauma to be a better reflection of outcome. Furthermore, because we are measuring on a process or organisational level, one can not specify to what changes the positive influence in outcome can be attributed.

In conclusion, when comparing the cohort of the year 2014 to the cohort of 2004–2005, one can see a marked increase in the mean age of the severely injured patient, though trauma scores remain comparable. The total number of acute interventions declines, mainly the number of trauma surgical interventions, the number of acute angio interventions, however, significantly increased. In light of all these changes, the mortality significantly declined for the most recent cohort; furthermore, the observed survival was better than the predicted survival. Showing improved trauma care in our hospital, in favour of the second period.
